# Calibration of discrete meta-parameters of bamboo flour based on magnitude analysis and BP neural network

**DOI:** 10.1371/journal.pone.0308019

**Published:** 2024-10-22

**Authors:** Lintao Chen, Rui Chen, Xiangwei Mou, Zhaoxiang Liu, Xu Ma, Xifeng Wu, Xiangwu Deng

**Affiliations:** 1 Department of Mechanical Engineering, Guangxi Normal University, Guilin, China; 2 College of Engineering, South China Agricultural University, Guangzhou, China; 3 College of Electronic Information Engineering, Guangdong University of Petrochemical Technology, Maoming, China; University of Sharjah, UNITED ARAB EMIRATES

## Abstract

In the research and development of technology and equipment for bamboo products deep processing, such as filling, drying, and medicinal use of bamboo flour (BF), the poor compaction and fluidity of BF materials entails the need for accurate discrete element model (DEM) and BF parameters to provide a reference for the simulation of BF processing operationsand the development of related equipment. The average particle size of the 5 types of BFs ranges from 0.136 mm to 0.293 mm, and the small particle size of BF particles causes to the number of BF particles in bamboo processing equipment to reach tens of millions or even billions. When conventional methods are used for simulation, ordinary computers cannot provide the required computing power. To address the aforementioned challenges, this paper proposes a calibration method for the discrete element contact parameters of BFs based on dimensional analysis and a back propagation (BP) neural network. Using particle scaling theory and dimensional analysis methods, the average particle size of the BF was increased to 1 mm, and the main discrete element contact parameters of the five types of BF to be tested were used as input layers. The injection method and sidewall collapse method were used to obtain the angle of repose (AR) as the output layer. Fifty groups were randomly selected using MATLAB for EDEM simulation, and the simulation results were trained using the BP neural network algorithm; an ideal neural network model was obtained, the discrete element parameters of different BFs were predicted, and physical experiments were performed to verify two types of AR and mold hole compression under calibrated parameters. The relative error between the simulated AR obtained through calibration parameters and the physical experimental values is less than 2.3%. Through BF parameter validity verification, the simulated maximum compression displacement and compression ratio after stabilization were 34.81 mm and 0.477, which were close to the actual experimental results of 34.77 mm and 0.461, respectively, verifying the accuracy of the neural network prediction model. The research results provide a reference for the simulation of BF processing operations and the development of related equipment.

## 1 Introduction

Because bamboo has strong adaptability and low requirements for its growth environment, it is widely distributed in China. Bamboo is a good raw material, because it is a perennial plant with a short growth cycle. Therefore, bamboo can be used as a resource in multiple fields, such as biomass fuel, food additives, fertilizers, etc. It occupies an important market position in industries such as food, furniture, and artificial boards that use bamboo flour (BF) as a raw material [1, 2]. In the processing, storage, and application of biomass materials such as bamboo, it is necessary to rely on appropriate processing equipment, and accurate contact parameters of BF models are the key to achieving mechanical processing of BF biomass materials. At the same time, these parameters are also necessary for the development and optimization of related equipment. The physical, mechanical, and chemical properties of biomass materials are affected by particle-related shapes, fiber properties, and viscosity, making it difficult for traditional methods to accurately obtain the contact parameters between processing equipment and biomass materials [3, 4].

The discrete element method (DEM) is an important method for designing, modeling, and studying particle systems and can effectively simulate particle motion during biomass material screening, crushing, and transportation [5]. Scholars at home and abroad have used the DEM to conduct relevant research on different particle material parameters, including intrinsic parameters and contact parameters [6, 7]. Domestic and foreign scholars have studied the calibration of contact parameters, including those for rice, soil, and straw [8]. Zhang Guozhong et al. conducted a study on the calibration of discrete element parameters for water chestnut [9]; Song Zhanhua et al. used a combination of experimental and simulation methods to calibrate soil contact parameters [10]; Yuan Quanchun et al. calibrated the discrete element parameters of organic fertilizers [11]; Wang Weiwei et al. calibrated the discrete element parameters for dense molding of corn straw powder [12]; and Lu Fangyuan et al. calibrated the discrete element parameters of rice sprouts under different moisture contents [13]. There are many discrete element simulation parameters, which are generally obtained through direct measurement and virtual calibration [14–17], but there are still few reports on the calibration of BF discrete element parameters.

Due to the small particle size of BFs, the compactness and fluidity of the material are poor. The number of BF particles in bamboo product processing equipment can reach tens of millions or even billions. Because of the limited capacity of ordinary computers, the particle scaling method is currently a feasible approach. Scholars have conducted discrete element parameter calibration research on wheat flour [18], coal [19] and lime [20, 21].

The existing calibration methods mainly involve trial-and-error methods, sequential calibration methods, and experimental design methods [21]. There is a high degree of nonlinearity between the micromechanical parameters and the macromechanical parameters of the DEM, and there is significant randomness in manual parameter calibration methods, resulting in poor reproducibility of the experimental results [22, 23]. In engineering applications, back propagation (BP) neural networks are usually established to express nonlinear systems [24]. The macroscopic parameters of the corn straw uniaxial creep test were input [25] into the trained neural network for microscopic parameter calibration. The BP neural network model was constructed [26] with particle microparameters as the input and rock and soil shear strength indicators as the outputs, and a biaxial compression numerical model parallel testing technology was developed to accelerate the process of obtaining neural network sample data. A starch discrete element contact parameter calibration method for a BP neural network model was established [27]. The BP neural network model is a multilayer feedforward neural network. When the signal is passed forward and forward, the error will propagate back. Throughout the process, the network weights and thresholds are continuously adjusted based on the propagation error, so that the output value of the neural network model continuously approaches the expected value [28, 29].

In summary, in response to the lack of accurate discrete element models and parameters in the research and development of technologies and equipment for bamboo product deep processing, such as filling, drying, and medicinal use of BF, this study proposes a BF discrete element contact parameter calibration method based on dimensional analysis and BP neural network. Using particle scaling theory and dimensional analysis methods, the average particle size of BFs was increased to 1 mm. The contact parameters of the five types of discrete BF elements to be tested were used as input layers, and the angle of repose (AR) was obtained from two measurement methods as the output layers. Fifty groups were randomly selected using MATLAB for EDEM simulation. The BP neural network algorithm was used to train the simulation results, and an ideal neural network model was obtained to predict the parameters of different BF discrete elements. Physical experiments were performed to verify two types of a AR and mold hole compression under calibrated parameters. This study provides a reference for the simulation of BF processing operations and the development of related equipment.

## 2 Materials and methods

### 2.1 Bamboo flour acquisition

**Fig 1:**
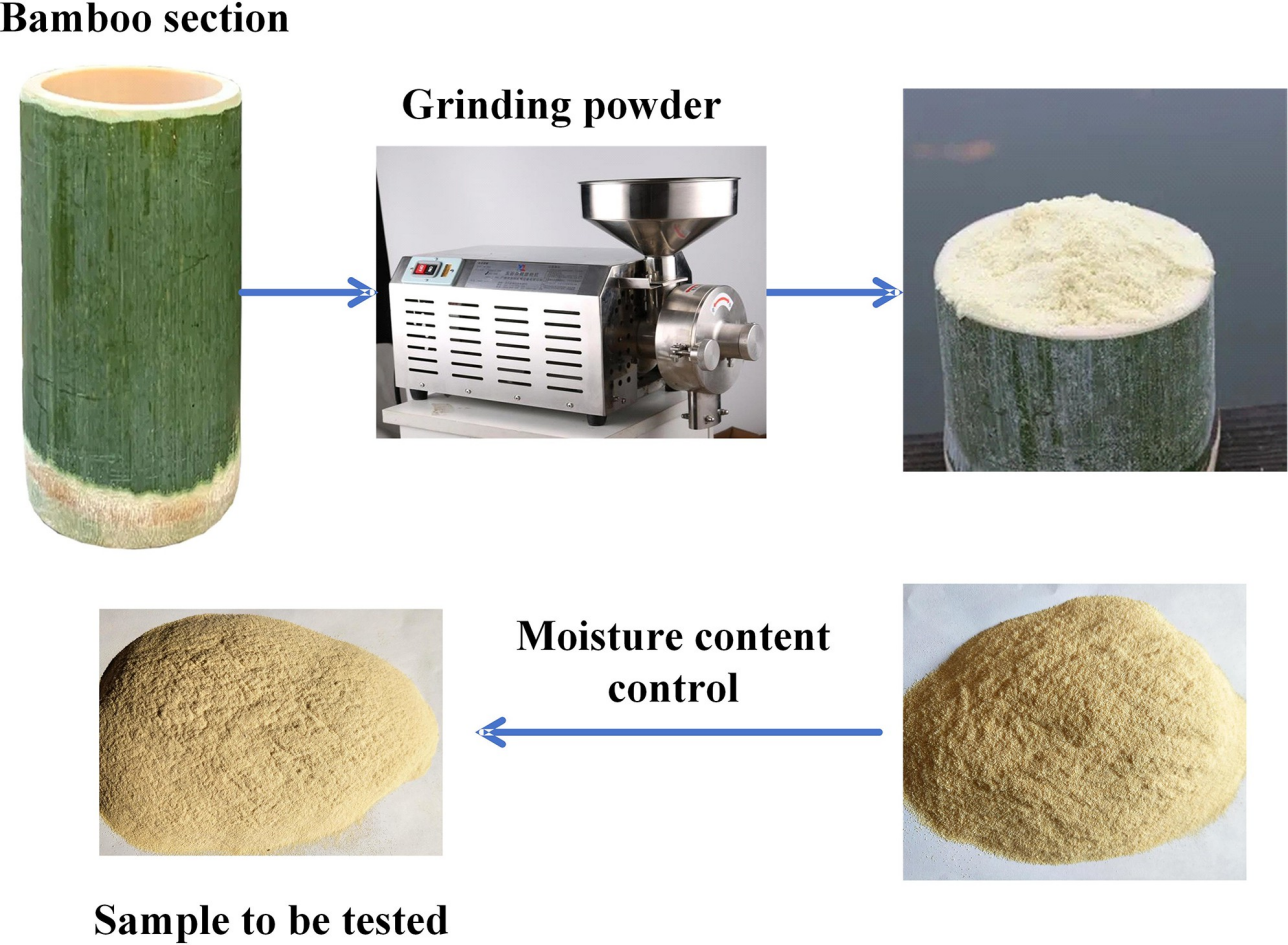
Raw materials obtained from BFs.

As shown in Fig 1, the BF test samples were collected through a forest farm under the jurisdiction of the Science and Technology Bureau of Resource County, Guilin, Guangxi Zhuang Autonomous Region (bordered by 110°13′~110°54′ E and 25°48′~26°16′ N). The BF test samples were arrow BF of *Z*_1_, fine powder BF of *Z*_2_, palm BF of *Z*_3_, high section BF of *Z*_4_, and giant dragon BF of *Z*_5_, all of which were aged 3–5 years and had a diameter at breast height of 11.7 cm. The static bending strength is 169.78 MPa, and the compressive strength is 72.35 MPa. The middle section of the bamboo test sample, which contains green and yellow, bamboo, was ground to a moisture content below 10%, and the powder was ground to obtain the test sample. The experiment was conducted at the Mechanical Engineering Experimental Center of Guilin Guangxi Normal University. To determine the density of the BF, a 350 mL measuring cylinder was used, and the BF was added to the cylinder with a spoon. The pile density was calculated for every 55 mL of BF added, and this process was repeated 5 times. Finally, the average pile density of the BF was 680 kg∙m^-3^. Ignoring the influence of gaps in the BF, the measured density of the BF piles is approximately equal to the particle density.

To obtain the average particle size of the BFs, standard inspection sieves with mesh sizes of 0.380, 0.198, 0.106, and 0.048 mm were used for screening [18]. A total of 450 g of each type of BF was weighed and placed on a top sieve. The sieve it was shaken back and forth. Finally, the mass of BF in each layer of the sieve was weighed. Taking arrow BFs and palm BFs as examples, the particle size distributions of different types of BF particles were obtained, as shown in Tables 1 and 2.

**Table 1:** Particle size distribution of arrow BF.

Serial Number	Particle size /mm	Mass /%
1	>0.380	5.54
2	0.199~0.380	3.25
3	0.107~0.198	31.19
4	0.049~0.106	56.36
5	≤0.048	3.66

**Table 2:** Particle size distribution of palm BF.

Serial Number	Particle size /mm	Mass /%
1	>0.380	6.85
2	0.199~0.380	5.25
3	0.107~0.198	29.91
4	0.049~0.106	55.32
5	≤0.048	2.67

The analysis revealed that more than 85% of the measured BF particles were between 0.049~0.198 mm in size. The average particle size is calculated based on mass, and the formula is as follows:

$$D_{Z}=\sum_{i=1}^{n}m_{i}d_{i}$$ (1)

where *D*_Z_ is the average particle size of the BF, m; *m*_i_ is the mass fraction of particles in the *i*-th grade, %; and *d*_i_ is the median particle size of particles in the *i*-th grade, m.

By substituting the data in the table into Eq (1), the average particle size of arrow BF is 0.167 mm, and the average particle size of palm BF is 0.156 mm. Similarly, the average particle sizes of the fine powder BFs, high section BFs, and giant dragon BFs were 0.136, 0.212, and 0.293 mm, respectively. The average particle size of the 5 types of BFs ranged from 0.136 mm to 0.293 mm. To simplify the parameter settings, they were all simplified into spherical shapes with a diameter of 1 mm in the discrete element software.

### 2.2 Particle scaling principle

#### 2.2.1 Dimensional analysis theory

The DEM is an effective solution for calibrating seed particle parameters. Although DEM has made significant progress in recent years, computer computing power still faces significant challenges. Computing power is closely related to the size and number of particles, and the smaller the particle size and the more particles there are, the greater the computational workload.

This study selected the similarity theory of discrete element systems to handle particles and manually scaled them. Based on similarity theory and dimensional analysis, combined with practical engineering experience, the accuracy of using the particle scaling method for simulation experiments of simulated particles is clarified.

To enable reasonable and effective simulation of the DEM, the simulation parameters of the DEM are adjusted to ensure that the scaled particle simulation results exhibit the same dynamic and static characteristics as the original system particles as much as possible, and to reduce simulation errors caused by particle scaling. Therefore, the scaling factor between the original system physical model and the individual physical quantities within the scale model was established [18].

$$\bar{q}=\lambda_{q}\cdot q$$ (2)

where *q* is any parameter in a physical system; *λ*_*q*_ is Scale; and $\bar{q}$ is scaling any parameter in the system.

When all physical quantity scaling factors are determined, a scaling model is established. For the original system, except for a few independent fundamental quantities, all other physical quantities can be calculated through fundamental quantities. If length [*L*], density [*ρ*], and time [*T*] are selected as basic quantities to derive scaling relationships for other physical quantities, the scaling factor for basic quantities is *λ*_*L*_ = *h*; *λ*_*T*_ = *h*; *λ*_*ρ*_ = 1.

From this, it can be inferred that the proportion factor of Young’s modulus *E* and velocity *v* is:

$$F=EAu/L\Rightarrow [E]=[e]{\left[L\right]}^{2}{\left[T\right]}^{2}\Rightarrow \lambda_{E}=\lambda_{\rho }{\lambda_{L}}^{2}{\lambda_{T}}^{2}=1$$ (3)

shere *F* is the axial force, N; *A* is the cross sectional area, m^2^; and *u* is the axial displacement, m.

$$v=L/T\Rightarrow [v]=[L]{\left[T\right]}^{-1}\Rightarrow \lambda_{v}=\lambda_{L}{\lambda_{T}}^{-1}=1$$ (4)

where *L* is the length, m; and *T* is the time, s.

**Fig 2:**
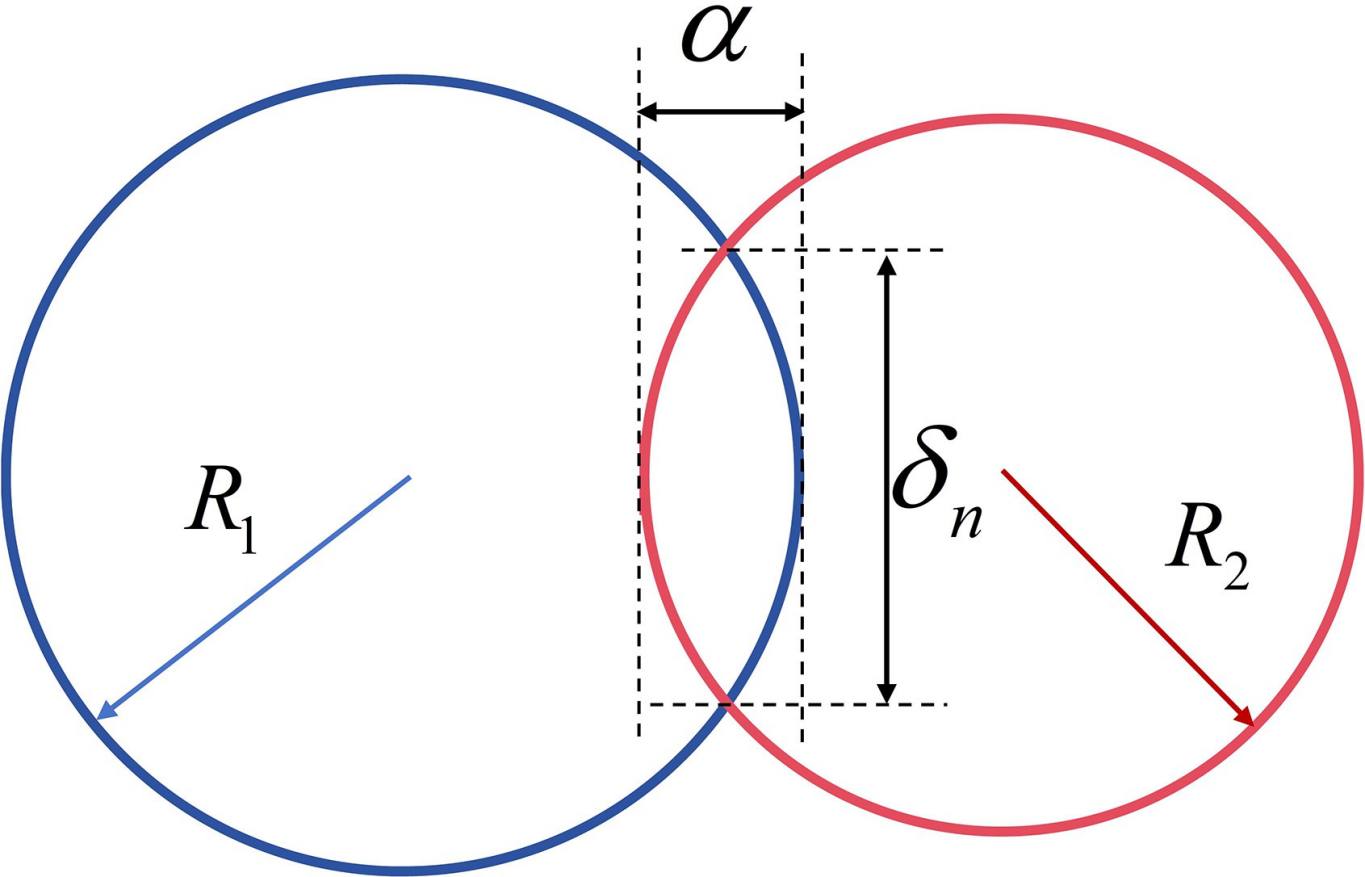
Schematic diagram of the JKR bonding model.

For the adhesive elastic particle model, the contact force between two adhesive (Fig 2) elastic spheres is:

$$F_{n}=\frac{4E^{*}a^{3}}{3R^{*}}-\sqrt{8\pi \Delta \gamma E^{*}a^{3}}$$ (5)

where *F*_n_ is contact normal force, N; E* is the effective elastic modulus, Pa; *a* is the contact surface radius between particles, m; R* is the effective radius of particles, m; and Δ*γ* is the surface free energy of the particles, J/m^2^.

From the adhesive force $\sqrt{8\pi \Delta \gamma E^{*}a^{3}}$ between particles, converting the formula to the stress‒strain form indicates that the second term does not exhibit scale invariance. Therefore, the JKR model is non scale invariant and does not meet the interaction conditions of scaling models.

To meet the scale invariance of the model, scaling *k* to *k*_h_ will result in a scaled linear contact model, which is scale invariant.

$$\bar{k}=k_{h}$$ (6)

where $\bar{k}$ is the scale stiffness of the model, N/m, and *k* is the model stiffness, N/m.

$$\left\{ \begin{array}{l} k=[\rho ]{\left[L\right]}^{3}{\left[T\right]}^{2}\Rightarrow \lambda_{k}=h\Rightarrow \lambda_{\bar{k}}=h^{2}\\ \bar{E}=L\bar{k}u/A\Rightarrow [E]=[L]\bar{k}{\left[L\right]}^{-2}\Rightarrow \lambda_{\bar{E}}=h \end{array}\right.$$ (7)

where $\bar{E}$ is the scaling Young’s modulus of the model, Pa.

The parameter elastic modulus is not a fixed original value when the density of the scaled model remains constant, and the contact stiffness is linearly related to the particle size and is not a fixed constant. This is scaled as the diameter changes, where the larger value is taken based on the range of the parameter.

#### 2.2.2 Scaling contact principle

According to Newton’s laws of motion, the differential equation for the normal overlap between particles is as follows:

$$m_{e}\delta_{n}^{n}=k_{n}\delta_{n}+c_{n}\delta_{n}^{'}$$ (8)

where *m*_e_ is the effective mass, kg; *k*_n_ is the particle stiffness, N/m; *c*_n_ is the damping coefficient, kg/s; and *δ*_n_ is the overlap amount, m.

$$m_{e}=\frac{4\pi R_{i}^{3}\rho \beta^{3}}{3(1+\beta^{3})}$$ (9)

where *ρ* is the particle density, kg/m^3^; *β* is the particle size ratio; and *R*_i_ is the original system particle radius, m.

$$R_{e}=\frac{R_{i}R_{j}}{\left(R_{i}+R_{j}\right)}=\frac{R_{i}\beta }{1+\beta }$$ (10)

where *R*_e_ is the effective radius, m, and *R*_*j*_ is the scaling system particle radius, m.

We convert public announcements (9) and (10) into dimensionless quantities:

$$\delta_{n}^{*}=\delta_{n}/R;\;\delta_{n}^{'*}=\delta_{n}^{'}/v_{0};t^{*}=t/(R_{i}/v_{0})\Rightarrow \delta_{n}^{''*}=\delta_{n}^{''}/(R_{i}/v_{0}^{2})$$ (11)

where * is a dimensionless number; t is time, s; and *v*_0_ is speed, m/s.

Substituting into Eq (10) yields:

$$\frac{4\pi R_{i}^{3}\rho \beta^{3}v_{0}^{2}}{3(1+\beta^{3})}\delta_{n}^{''*}=k_{n}R_{i}\delta_{n}^{*}+c_{n}v_{0}\delta_{n}^{'*}$$ (12)

where *k*_n_ is the particle stiffness, N/m, and *c*_n_ is the damping coefficient, kg/s.

Simplification can lead to:

$$\frac{4\pi \beta^{3}}{3(1+\beta^{3})}\delta_{n}^{''*}=\frac{k_{n}\delta_{n}^{*}}{R_{i}\rho v_{0}^{2}}+\frac{c_{n}\delta_{n}^{'*}}{R_{i}^{2}\rho v_{0}}$$ (13)

The coefficients in Eq (13) can be represented by the following dimensionless numbers:

$$\pi_{1}=\frac{4\pi \beta^{3}}{3(1+\beta^{3})};\;\pi_{2}=\frac{k_{n}}{R_{i}\rho v_{0}^{2}};\;\pi_{3}=\frac{c_{n}}{R_{i}^{2}\rho v_{0}}$$ (14)

In a particle scaling system, the relative overlap between particles remains constant, and *π*_1_ is equal to *λ*_1_ as a constant, indicating the need to ensure that the particle size ratio remains constant; *π*_2_ is equal to *λ*_2_ as a constant, where *k*_n_ is equal to *R*_i_ as a constant, verifying the linear variation in the stiffness and radius; and *π*_3_ is equal to *λ*_3_ as a constant, where *c*_n_ is equal to $R_{i}^{2}$ as a constant. These results indicated the proportional relationship between the recovery coefficient and the square of the radius.

The van der Waals force is the main source of BF adhesion, and the study uses a theoretical adhesive elasticity model to represent the van der Waals force [18].

For the “Hertz–Mindlin with Johnson–Kendall–Roberts (JKR)” bonding model, van der Waals forces are associated with the particle radius:

$$f_{\mathrm{JKR}}=-3\pi \gamma R^{*}$$ (15)

where *γ* is the surface energy of per unit contact area, J/m^2^.

The relationship between the tensile strength and indirect particle contact force of a hard monodisperse sphere system with randomly isotropic materials was provided by [18].

$$f=\frac{4\pi R^{2}}{\phi k_{1}}\sigma$$ (16)

where *f* is the contact force between particles, N; *ϕ* is the fill rate, %; *k*_1_ is the coordination number, N; and *σ* is the tensile strength, MPa.

The analysis revealed shows that the indirect contact force of the BF is directly proportional to the square of the scaled particle radius. As the particle radius of the BF increases, the contact surface area between individual pairs of particles also increases. The correlation between the adhesion force and the contact area between particles, which is proportional to the square of the particle radius, indicates a quadratic relationship between the adhesion force and the particle radius. With the “Hertz–Mindlin with JKR” model setting, the JKR varies with the scaling ratio and has no fixed reference, which needs to be determined through calibration according to the range. By using the scaling factor of physical quantities, the relationship between the simulated physical quantities and actual physical quantities of the particles can be determined. Using large simulated particles instead of actual small particles can effectively shorten the calculation time. However, because the object of this article has 5 different particle sizes of BF, there are also certain differences in magnification. Based on the particle size distribution of the BF (0.136 mm—0.293 mm), similarity theory, and dimensional analysis mentioned in the previous 5 sections, to facilitate the parameter setting of the simulation and minimize simulation errors and obtain an effective simulation time [18–20], the BF particles were simplified into round balls with a diameter of 1 mm for simulation.

### 2.3 Method for measuring the angle of repose

#### 2.3.1 Injection method

**Fig 3:**
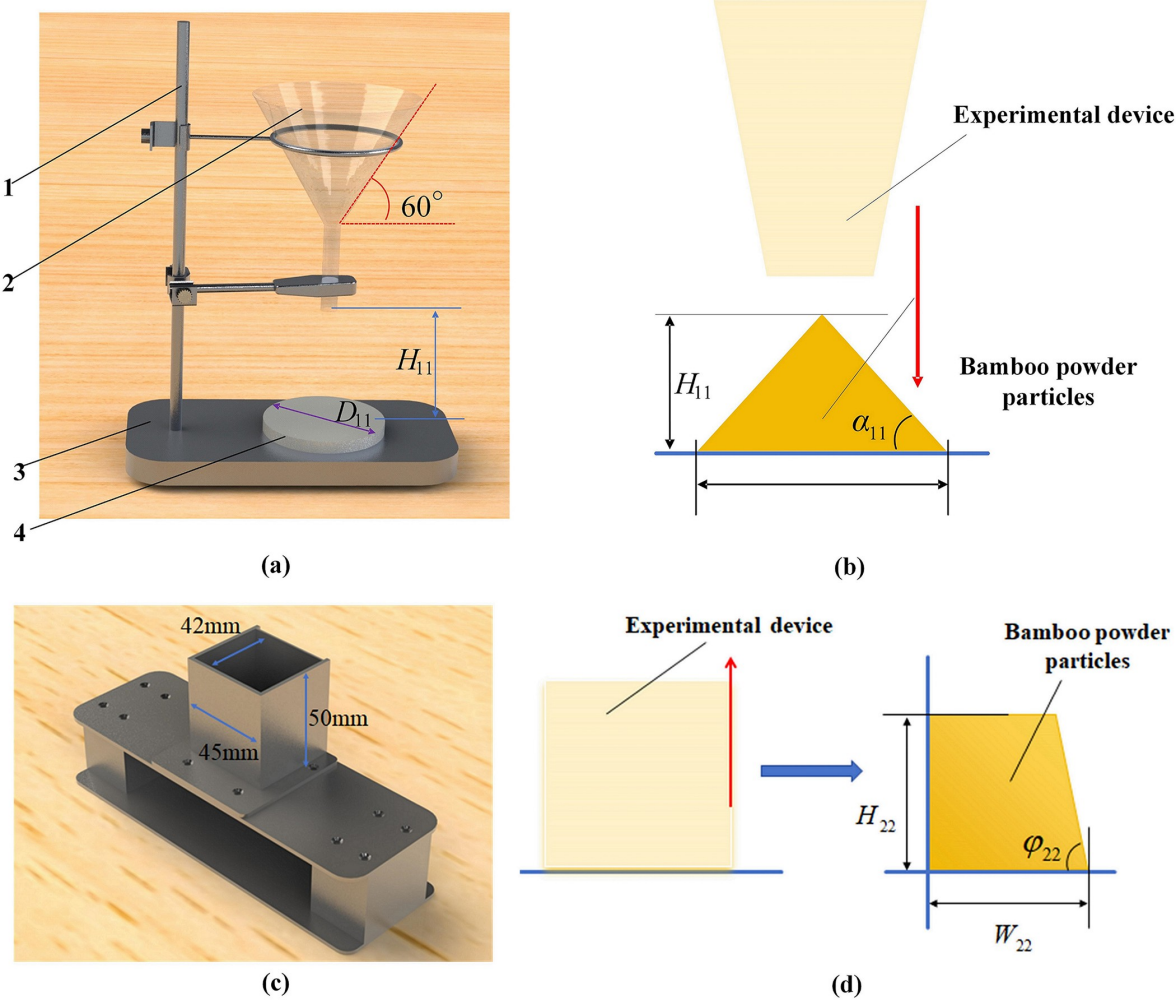
Physical measurement method for the angle of repose. (a) The device for measuring the angle of repose by the injection method; (b) The principle for measuring the angle of repose by the injection method; (c) The device for measuring the angle of repose by the sidewall collapse method; (d) The principle for measuring the angle of repose by the sidewall collapse method.

The injection method (IM) is used to measure the AR *α*_11_ for the BF, as shown in Fig 3(A) and 3(B) [18–20]. The diameter of the funnel discharge port is 10 mm, with a taper of 60°. The diameter of the stainless steel cylindrical chassis *D*_11_ is 90 mm, and the distance between the lower end of the funnel and the upper surface of the cylindrical chassis is 80 mm. The AR is calculated according to Eq (17). The average of the results was taken, and the experiment was repeated 5 times.


$$\alpha_{11}=\arctan\frac{2H_{11}}{D_{11}}$$ (17)

#### 2.3.2 Sidewall collapse method

The AR *φ*_22_ for the BF was measured using the sidewall collapse method (SCM) [13], as shown in Fig 3(C) and 3(D). The fixed mass BF was weighed and poured it into a stainless steel box without a top cover. The AR is calculated according to Eq (18).


$$\varphi_{22}=\arctan\frac{H_{22}}{W_{22}}$$ (18)

#### 2.3.3 Measurement results of AR

The IM is used to measure the AR α_11_ of the BF, and the SCM is used to measure the AR φ_22_ of the BF. The results of the determination of arrow BF Z_1_, fine powder BF Z_2_, palm BF Z_3_, high section BF Z_4_, and giant dragon Z_5_ are as follows (Table 3).

**Table 3:** Measurement parameters of five BF.

Type	*α*_11_	*φ*_22_
Z_1_	33.90	50.12
Z_2_	39.10	54.91
Z_3_	35.96	50.84
Z_4_	38.10	56.93
Z_5_	43.92	52.00

### 2.4 Simulation model and sample construction

#### 2.4.1 Contact model selection

The “Hertz–Mindlin with JKR” bonding model is based on the Hertz theoretical particle model and is suitable for simulating materials with significant bonding and agglomeration between particles due to static electricity, fibers, and moisture [18–22]. Therefore, the contact model was chosen for the discrete element parameter calibration study of the BF.

#### 2.4.2 Selection of simulation parameters

Based on the discrete element parameters of powder and stainless steel plates in domestic and foreign literature, as well as the generic EDEM material model database (GEMM) built into EDEM [22], the parameter settings are shown in Table 4. Here, *x*_1_ to *x*_7_ are the range values, and there are uncertainties due to the diversity of BF particle shapes and sizes, surface roughnesses, environmental factors (such as temperature and humidity), and uneven material properties. Therefore, the parameters may vary in practical situations, and the range of values can more comprehensively and accurately reflect the actual performance of the BF, thereby improving the reliability and applicability of the simulation results.

**Table 4:** Parameters required in DEM simulation.

No	Simulation parameters	Numerical value
1	Particle radius /mm	0.50
2	Poisson’s ratio of BF	0.25
3	BF shear modulus /Pa	6.50×10^7^
4	BF density /kg∙m-3	6.80×10^2^
5	Poisson’s ratio of stainless steel plate	0.30
6	Shear modulus of stainless steel plate /Pa	7.00×10^10^
7	Density of stainless steel plate /kg∙m^-3^	7.80×10^3^
8	BF–BF recovery coefficient *x*_1_	0.15~0.55
9	Static friction coefficient between BF and BF *x*_2_	0.20~0.80^A^
10	Rolling friction coefficient between BF and BF *x*_3_	0.05~0.35^A^
11	Recovery coefficient of BF stainless steel plate *x*_4_	0.15~0.80^A^
12	Static friction coefficient between BF and stainless steel plate *x*_5_	0.20~0.70^A^
13	Rolling friction coefficient between BF and stainless steel plate *x*_6_	0.10~0.80^A^
14	Surface energy *x*_7_/J∙m^-2^	0.01~0.05^A^

Note: The superscript A represents the experimental variable.

#### 2.4.3 Discrete element simulation of the angle of repose

Two models of the AR measurement devices were established and placed in EDEM according to the parameters in Table 3. The dynamic particles of the BF was randomly generated, the mass of the BF was set to 45 g using IM, and the mass of the BF was set to 55 g using SCM.

**Fig 4:**
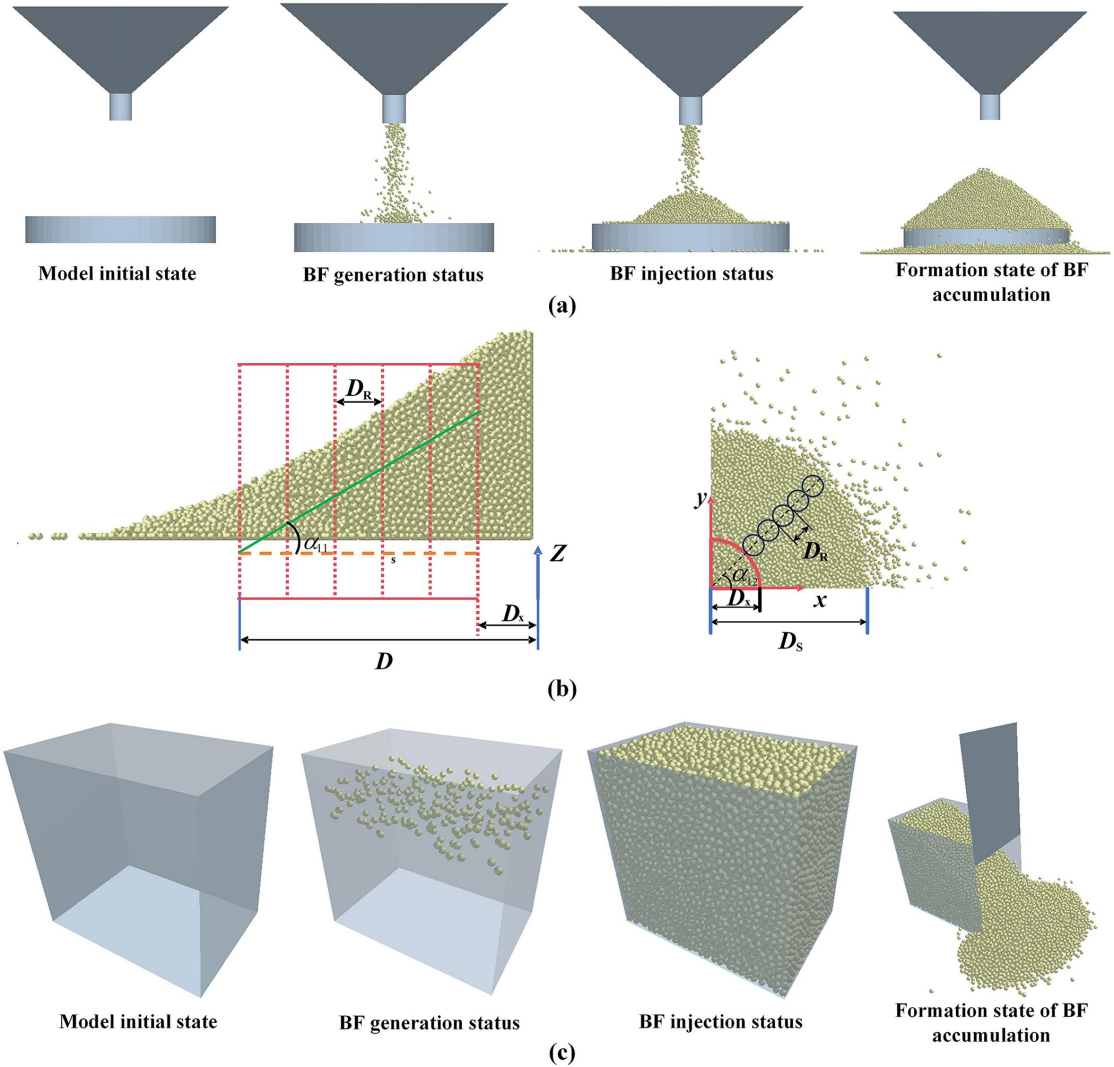
Discrete element simulation process for the angle of repose. (a) Simulation with the angle of repose measurement using the injection method; (b) schematic diagram of the method for measuring the angle of repose; (c) simulation of the angle of repose measurement using the sidewall collapse method.

1) Injection method: As shown in Fig 4A, the simulation time is 15 seconds. When the receiving particles on the bottom of the cylinder are in an overflow state, set the generation rate is set to 0, and the simulation is continued. After the particles in the funnel have finished falling, the software postprocessing function is used to record the particle position, the trend of the particle stacking height over time is recorded, the height under relative stillness is determined, and the AR of the BF is measured. To accurately measure the AR of the BF, the EDEMpy library developed based on Python was used to analyze the simulation results [20]. A radial cylindrical container array was defined to determine the highest BF particle in each container; linear fitting of the centroid coordinates of these BF particles was performed using the least squares method; the angle between the fitted line and the horizontal line was the AR. The measurement method is shown in Fig 4B.

For the best results, a region with stable stacking was selected for analysis. The lower limit value *D*_x_ of the analysis region is 10 mm, and the upper limit value *D*_s_ is 40 mm. The diameter *D*_R_ of the container is defined as 6 mm, where *α*_12_ is the slicing direction angle. The average AR was obtained by measuring multiple different directional angles.

2) Sidewall collapse method: As shown in Fig 4C, during the simulation process, the right surface of the material box is removed, and the material gradually collapses and slides down. The remaining material forms a stable slope in the rectangular body, ending the simulation and measuring the AR of the BF.

#### 2.4.4 Sample construction

Random sampling was conducted using MATLAB for the parameters that required recalibration, such as the BF-BF recovery coefficient *x*_1_, BF-BF powder static friction coefficient *x*_2_, BF-BF rolling friction coefficient *x*_3_, BF-stainless steel plate recovery coefficient *x*_4_, BF-stainless steel plate static friction coefficient *x*_5_, BF-stainless steel plate rolling friction coefficient *x*_6_, and surface energy *x*_7_. A total of 50 different combinations of discrete element parameters were designed (*x*_1_ to *x*_7_), as shown in Table 5. Discrete element simulation experiments were conducted based on the numerical combinations of the parameters to be calibrated, and the ARs corresponding to each set of parameters under the two methods were obtained.

**Table 5:** 50 sets of numerical simulation experimental results.

No	Discrete element simulation parameters								
	*x*_1_	*x*_2_	*x*_3_	*x*_4_	*x*_5_	*x*_6_	*x*_7_	*α*_11_	*φ*_22_
**1**	0.51	0.50	0.23	0.67	0.65	0.80	0.02	32.31	58.09
**2**	0.30	0.40	0.05	0.37	0.33	0.20	0.01	23.02	35.56
**3**	0.47	0.74	0.05	0.32	0.55	0.38	0.02	23.50	39.10
**4**	0.15	0.40	0.13	0.15	0.33	0.20	0.02	27.65	44.42
**5**	0.15	0.20	0.05	0.15	0.20	0.10	0.01	18.39	33.25
**6**	0.60	0.20	0.05	0.15	0.70	0.10	0.05	27.22	47.51
**7**	0.30	0.20	0.05	0.15	0.33	0.10	0.02	24.72	37.26
**8**	0.39	0.68	0.26	0.73	0.40	0.10	0.04	34.25	55.58
**9**	0.30	0.40	0.13	0.15	0.20	0.10	0.02	23.19	42.15
**10**	0.15	0.20	0.05	0.37	0.20	0.20	0.02	20.29	34.76
**11**	0.30	0.20	0.13	0.37	0.20	0.20	0.02	24.46	43.26
**12**	0.15	0.20	0.13	0.15	0.33	0.20	0.01	28.67	43.83
**13**	0.30	0.20	0.13	0.37	0.33	0.10	0.01	27.65	42.70
**14**	0.23	0.30	0.10	0.27	0.26	0.15	0.02	25.35	39.10
**15**	0.15	0.40	0.13	0.37	0.20	0.10	0.01	21.01	37.26
**16**	0.19	0.26	0.35	0.55	0.35	0.59	0.01	35.95	64.53
**17**	0.15	0.40	0.05	0.37	0.33	0.10	0.02	23.65	36.39
**18**	0.30	0.40	0.05	0.15	0.20	0.20	0.01	18.19	33.99
**19**	0.60	0.40	0.13	0.37	0.58	0.20	0.04	29.07	50.96
**20**	0.30	0.40	0.28	0.37	0.58	0.40	0.02	35.24	59.85
**21**	0.60	0.40	0.28	0.80	0.33	0.40	0.04	36.39	60.76
**22**	0.45	0.60	0.20	0.58	0.45	0.30	0.03	31.98	52.44
**23**	0.30	0.40	0.13	0.80	0.33	0.40	0.04	29.59	48.86
**24**	0.15	0.20	0.05	0.80	0.20	0.80	0.05	22.33	42.15
**25**	0.30	0.80	0.28	0.37	0.58	0.40	0.04	35.69	58.96
**26**	0.60	0.80	0.13	0.37	0.33	0.40	0.02	28.30	44.42
**27**	0.60	0.40	0.28	0.80	0.58	0.20	0.02	34.01	53.98
**28**	0.15	0.56	0.17	0.21	0.30	0.17	0.03	29.28	58.09
**29**	0.60	0.80	0.13	0.80	0.58	0.40	0.02	27.58	46.87
**30**	0.30	0.40	0.13	0.37	0.33	0.20	0.02	27.22	43.26
**31**	0.30	0.80	0.13	0.80	0.58	0.20	0.04	29.07	52.44
**32**	0.35	0.80	0.58	0.27	0.45	0.66	0.04	42.01	90.00
**33**	0.60	0.80	0.28	0.37	0.33	0.20	0.04	36.19	72.78
**34**	0.30	0.80	0.28	0.80	0.33	0.20	0.02	33.50	56.40
**35**	0.60	0.80	0.05	0.80	0.70	0.80	0.01	23.32	39.10
**36**	0.27	0.20	0.11	0.61	0.50	0.24	0.04	31.24	52.44
**37**	0.60	0.20	0.35	0.80	0.70	0.10	0.01	36.19	62.61
**38**	0.15	0.80	0.05	0.80	0.70	0.10	0.05	23.32	44.99
**39**	0.38	0.50	0.20	0.48	0.45	0.45	0.03	33.50	55.58
**40**	0.60	0.80	0.35	0.15	0.20	0.10	0.05	34.54	90.00
**41**	0.60	0.80	0.05	0.15	0.20	0.80	0.01	18.86	33.25
**42**	0.15	0.80	0.35	0.80	0.20	0.10	0.01	27.93	49.54
**43**	0.23	0.44	0.08	0.44	0.70	0.45	0.01	26.73	41.09
**44**	0.15	0.20	0.35	0.15	0.70	0.80	0.01	18.69	32.54
**45**	0.43	0.38	0.32	0.15	0.60	0.31	0.03	36.84	59.85
**46**	0.15	0.80	0.35	0.15	0.70	0.80	0.05	35.08	90.00
**47**	0.15	0.20	0.05	0.15	0.20	0.10	0.01	38.00	64.53
**48**	0.60	0.20	0.35	0.80	0.20	0.80	0.05	36.82	90.00
**49**	0.31	0.62	0.20	0.50	0.20	0.73	0.05	30.89	90.00
**50**	0.55	0.32	0.14	0.38	0.25	0.52	0.05	29.59	57.24

#### 2.4.5 BP neural network training

The BP neural network is the most widely used neural network that processes information by using a structure similar to the synapses of brain neurons [24, 25]. In BP neural networks, the more layers there are, the better the ability to fit the function. However, in reality, having more layers may lead to overfitting problems and increase training difficulty [24–27].

**Fig 5:**
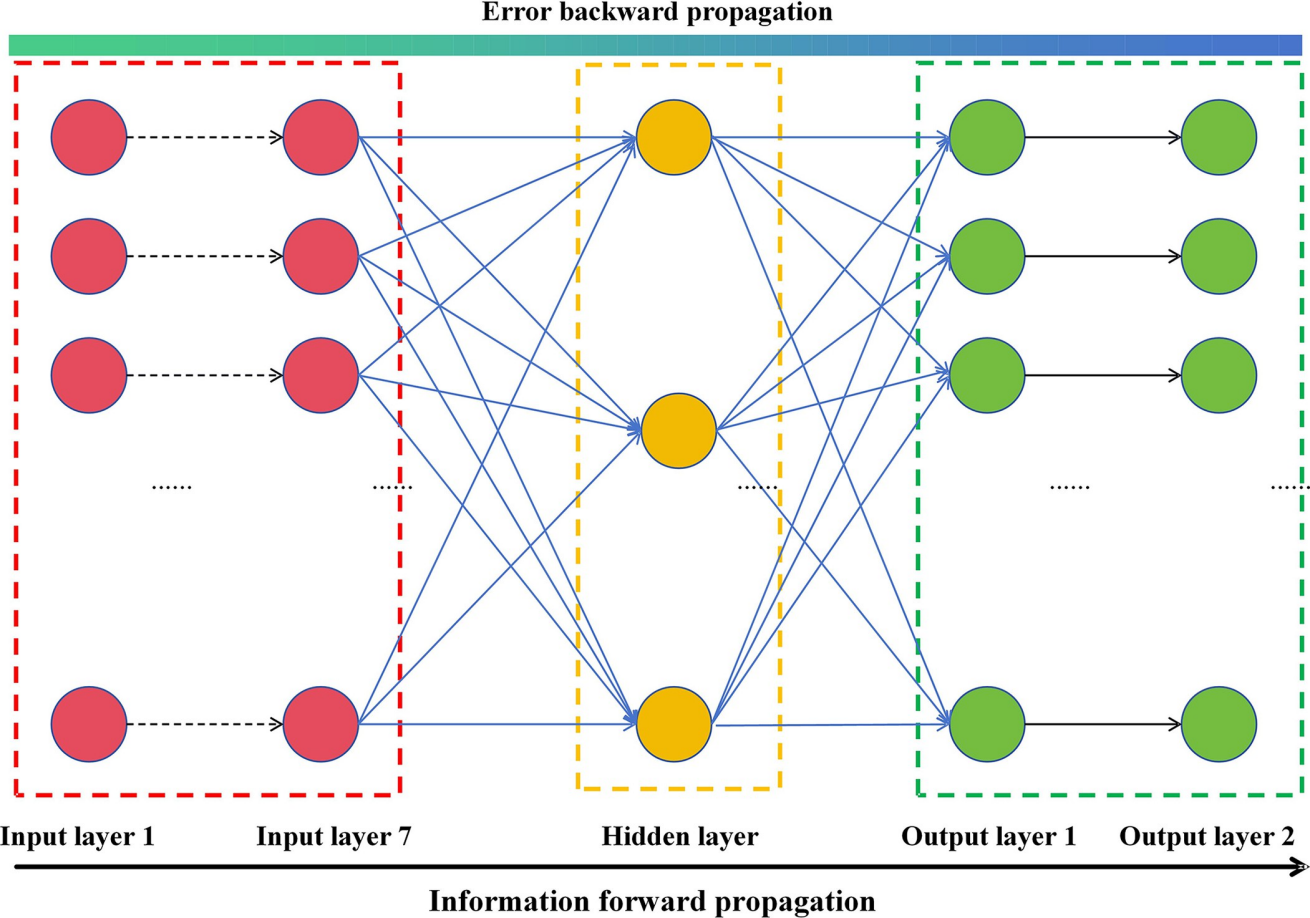
BP neural network topology structure.

This study used MATLAB to construct a network model with discrete element parameters to be calibrated as input layers, and the AR obtained from two methods as the output layers. As shown in Fig 5, both the number of neurons corresponding to the input layer and the number of neurons corresponding to the output layer were established based on the number of parameters required for the discrete element and the macroscopic AR behavior of the BF. The study randomly assigned 50 sets of data into three groups at percentages of 80%, 10%, and 10% for training, validation, and testing, respectively, of the BP neural networks and normalized all the data.

The training set loss function adopts the squared difference loss function, and the formula is as follows:

$$J(W,B)=\frac{1}{M}\sum_{I=1}^{M}[H_{\theta }{\left(X^{\left(I\right)}-Y^{\left(I\right)}\right]}^{2}+\frac{\lambda }{M}\sum_{l=1}^{L}W^{{\left[I\right]}^{2}}$$ (19)

where *J*(*W*, *B*) is the loss function; *M* is the number of experimental data samples; *H*_θ_(*X*^(I)^) is the model calculation value for the *I*-th sample; *Y*^(I)^ is the true value of the *I*-th sample; $\frac{\lambda }{M}\sum_{l=1}^{L}W^{{\left[I\right]}^{2}}$ is the regularization term; and *λ* is the regularization coefficient, which has a value of 1.

Because of the number of hidden neurons should be 2/3 of the input layer size plus 2/3 of the output layer size, the minimum value for hidden neurons is 6. The number of hidden neurons should be less than twice the size of the input layer, and the maximum number of hidden neurons should be 14. The appropriate number of neurons was selected by examining the impact of 6–14 neurons on fitting accuracy in the hidden layer. The expected output of the AR obtained from the two measurement methods is used, and the contact parameters of the simulated experimental AR results are predicted by constructing a BP neural network. A numerical simulation was performed using EDEM according to the method described in section "2.4.3", the relative error was calculated using Eq (20), and the accuracy of the predicted parameters was verified.

$$\sigma =\frac{\left|x_{t}-x_{s}\right|}{x_{t}}\times 100\%$$ (20)

where *σ* is the relative error, *x*_t_ is the experimental value, and *x*_s_ is the analog value.

## 3. Results and discussion

### 3.1 Analysis of the measurement results of the angle of repose

**Fig 6:**
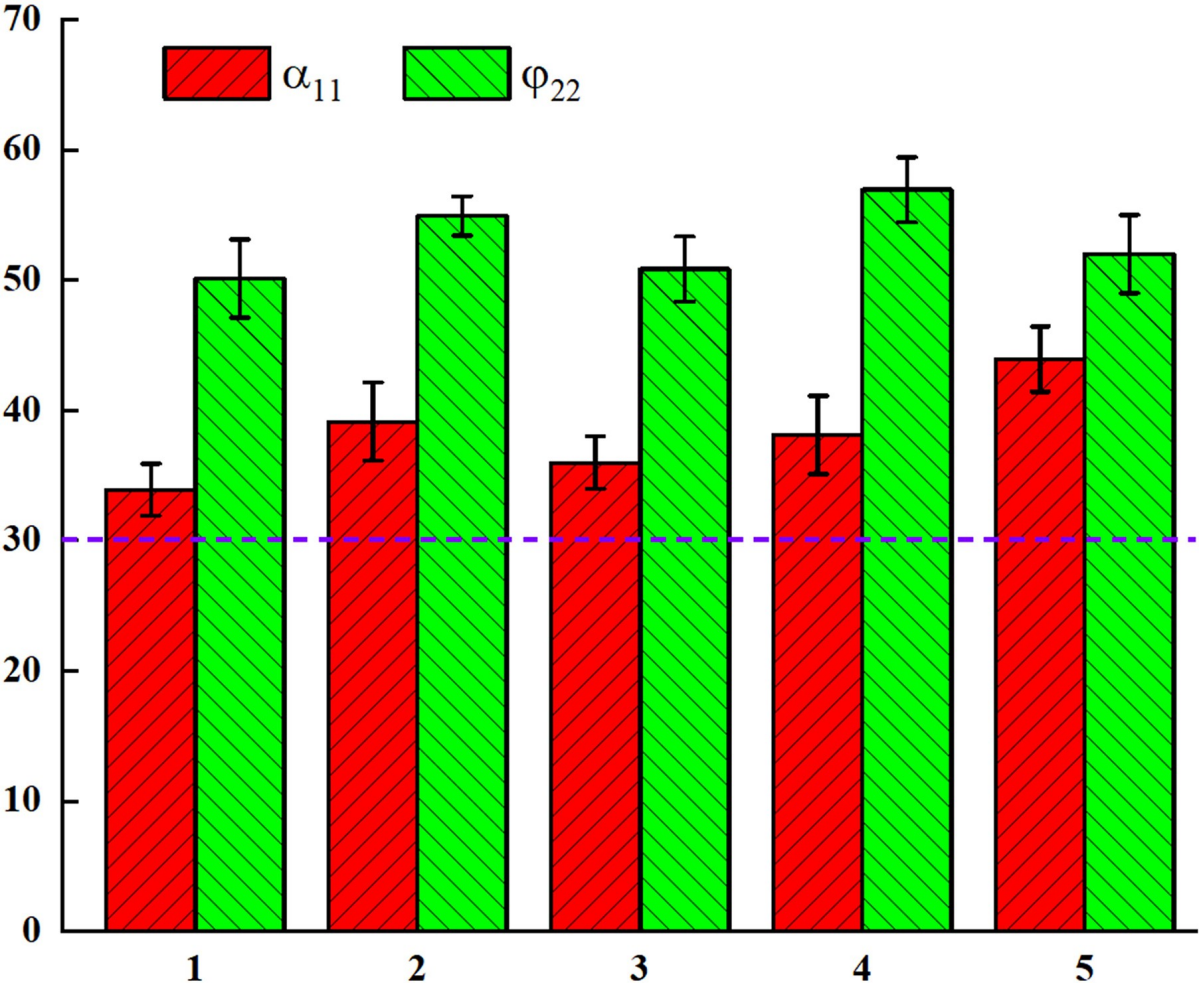
Analysis of the results of physical measurements with respect to the angle of repose of BF particles.

The ARs for the different types of BF are greater than 30°, as shown in Fig 6, indicating the poor fluidity of BF. The pairwise T-tests for different ARs showed that the P values were all greater than 0.05, indicating that there were differences in the ARs between different types of BF. This is because the particles have a certain degree of cohesion, and it can also be found that the ARs for the different BFs are *α*_11_<*φ*_22_. Both methods allow the particles in the measuring device to lose their binding ability and be discharged under gravity. However, compared with the SCM, the IM has a larger loss of support area for the BF which can lead to this phenomenon.

### 3.2 Discrete element result analysis

According to the experimental design established by random sampling in MATLAB, the IM and SCM were simulated, and two types of ARs were obtained. The results are shown in Table 5. The simulation also showed a rule of *α*_11_<*φ*_22_, which is consistent with the results of physical experiments, indicating that the Hertz–Mindlin with JKR model is suitable for this simulation study.

### 3.3 Analysis of the neural network training results

#### 3.3.1 Neural network model optimization

**Fig 7:**
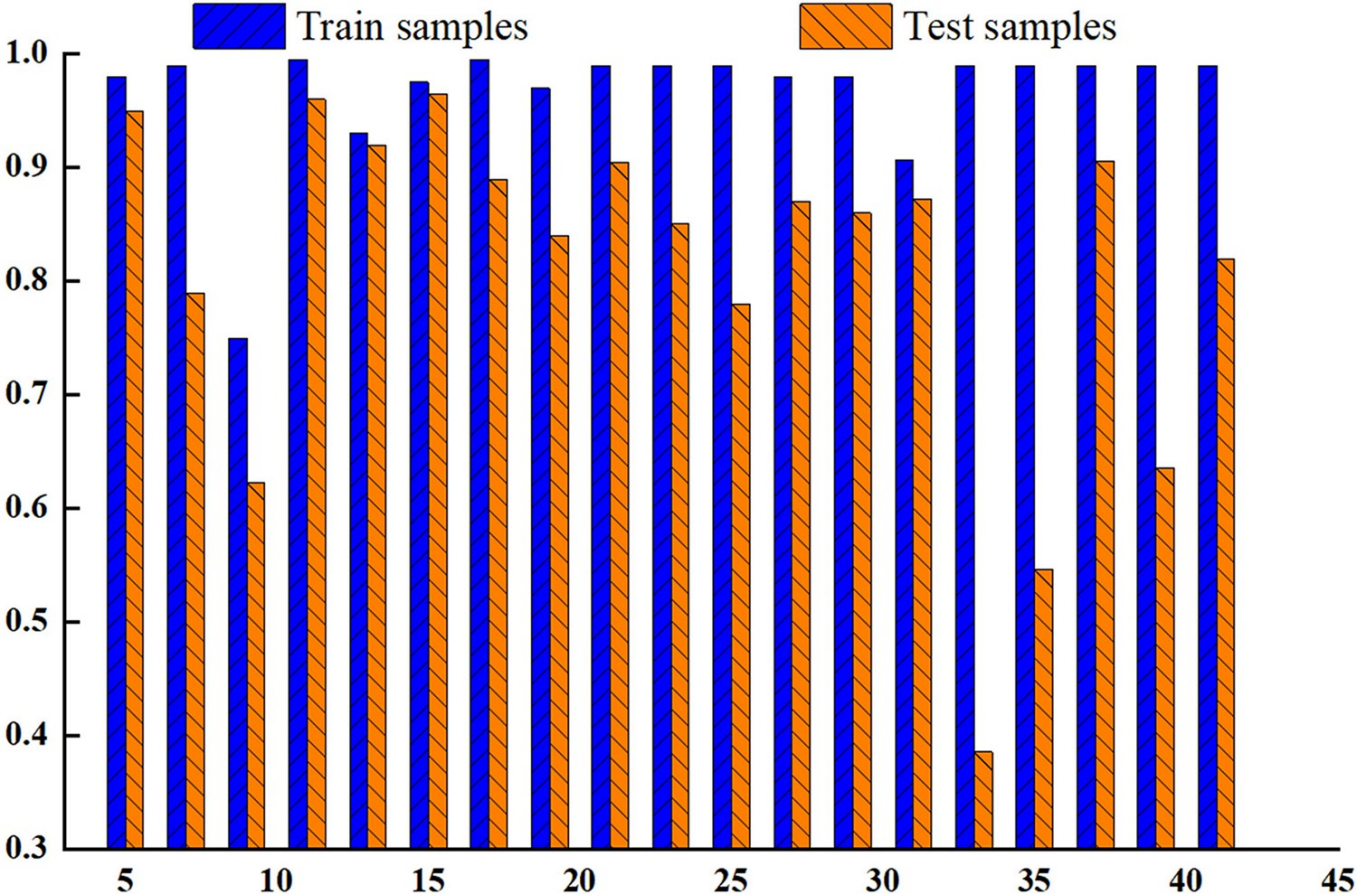
The influence of the number of neurons in the hidden layer on the determination coefficient.

Fig 7 shows the relationship between the number of hidden layer neurons and the determination coefficient in a neural network. The results show that when the number of neurons is 11, the determination coefficients of both the training and testing samples reach their maximum values. As the number of neurons increases [28, 29], the determination coefficient does not improve, and even oscillates. Although neural networks with fewer neurons have simple structures, this does not mean that the network’s ability to infer detailed information is lost. After repeated testing, the final number of hidden layer neurons in the BP neural network in this study was 11.

**Fig 8:**
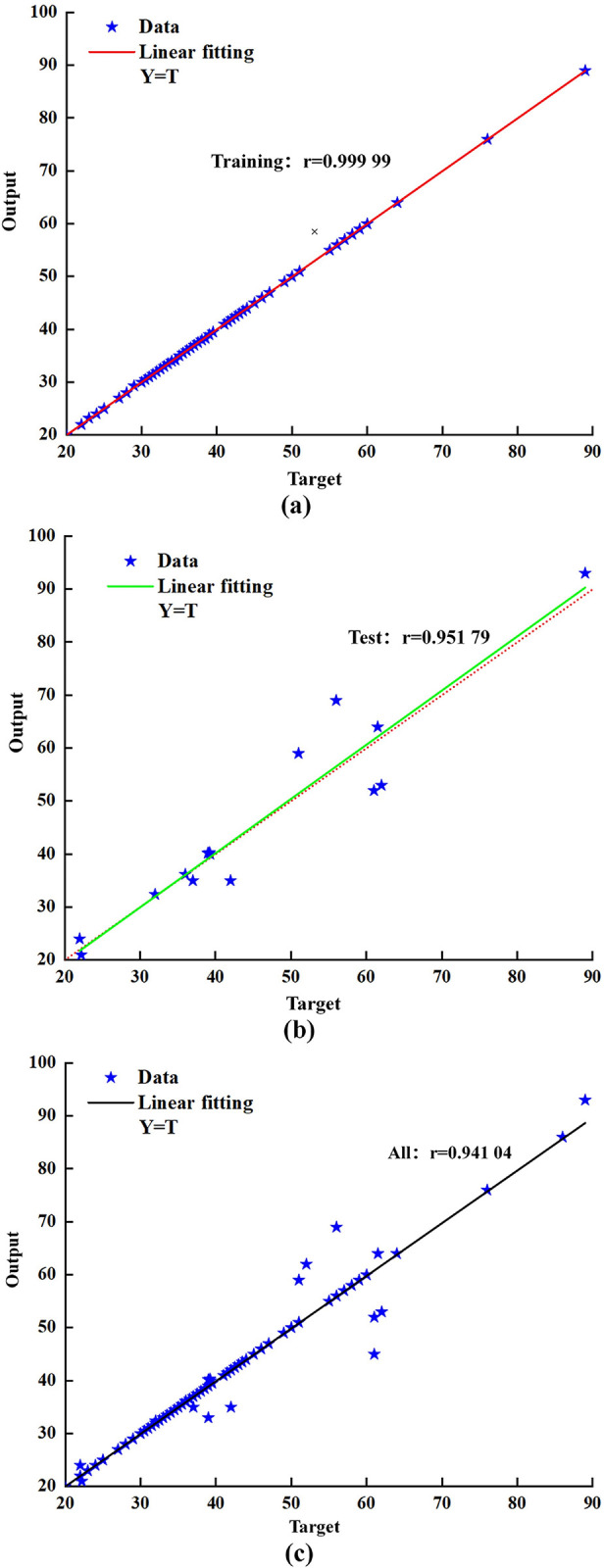
BP neural network linear regression analysis results. (a) Training sample; (b) test sample; (c) overall sample.

As shown in Fig 8, the BP neural network is trained, tested, and subjected to linear regression analysis of the predicted and target values of the overall sample. The results showed that the determination coefficients of the training samples, testing samples, and overall samples were all greater than 0.93, indicating that the established neural network has good prediction accuracy.

**Fig 9:**
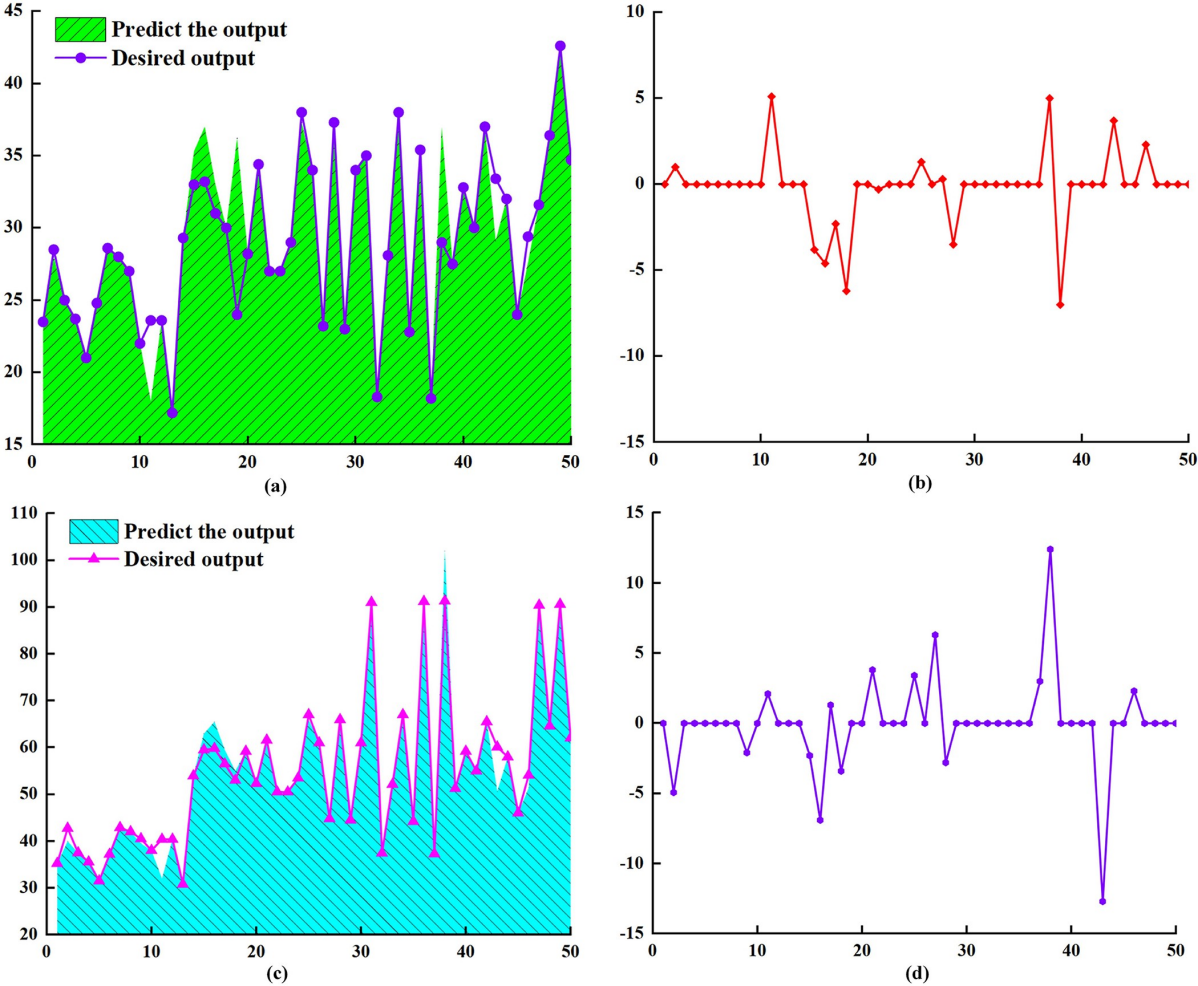
BP neural network prediction results and errors. (a) Injection method prediction results; (b) injection method error; (c) prediction results of the sidewall collapse method; (d) sidewall collapse method error.

Further analysis reveals that the prediction output of the neural network overlaps with the expected output as shown in Fig 9(A) and 9(C), indicating an error of 0, as shown in Fig 9(B) and 9(D). This proves that the constructed network has a high fitting ability, which is consistent with the results of the neural network regression analysis. Although the BP neural network has high fitting ability, the prediction error of certain sample points is relatively large. The accuracy of neural network prediction is closely related to the amount of training data, especially for a multi-input multi-output network. If sufficient training data are lacking, there may be significant errors in the network prediction values. To improve the accuracy of neural network prediction, increasing the sample capacity can be achieved.

#### 3.3.2 Neural network prediction

The constructed BP neural network was used to predict the discrete element parameters of different BFs, and the results are shown in Table 6. The contact parameters that affect particle behavior vary in different contact models or types of simulations, and corresponding calibration experiments should be selected based on the desired simulated particle behavior. This article uses bamboo segments in the middle of bamboo for grinding, controlling the moisture content to be less than 10%. This study has not yet conducted experiments on the effect of moisture content on simulated stacking angles.

**Table 6:** Contact parameters predicted by BP neural network model.

Type	x_1_	x_2_	x_3_	x_4_	x_5_	x_6_	x_7_
Z_1_	0.44	0.45	0.32	0.71	0.51	0.05	0.014
Z_2_	0.38	0.41	0.36	0.79	0.53	0.05	0.017
Z_3_	0.41	0.43	0.34	0.80	0.52	0.06	0.009
Z_4_	0.42	0.56	0.39	0.72	0.50	0.31	0.018
Z_5_	0.46	0.55	0.31	0.76	0.32	0.29	0.020

#### 3.3.3 Parameter validity verification

To verify the accuracy of the neural network prediction model, the validity of the discrete element parameters is verified. The first step is to compare the AR between the IM and the SCM for calibrating the discrete element contact parameters. Step 2 is to design compression experiments for BF mold holes.

**Fig 10:**
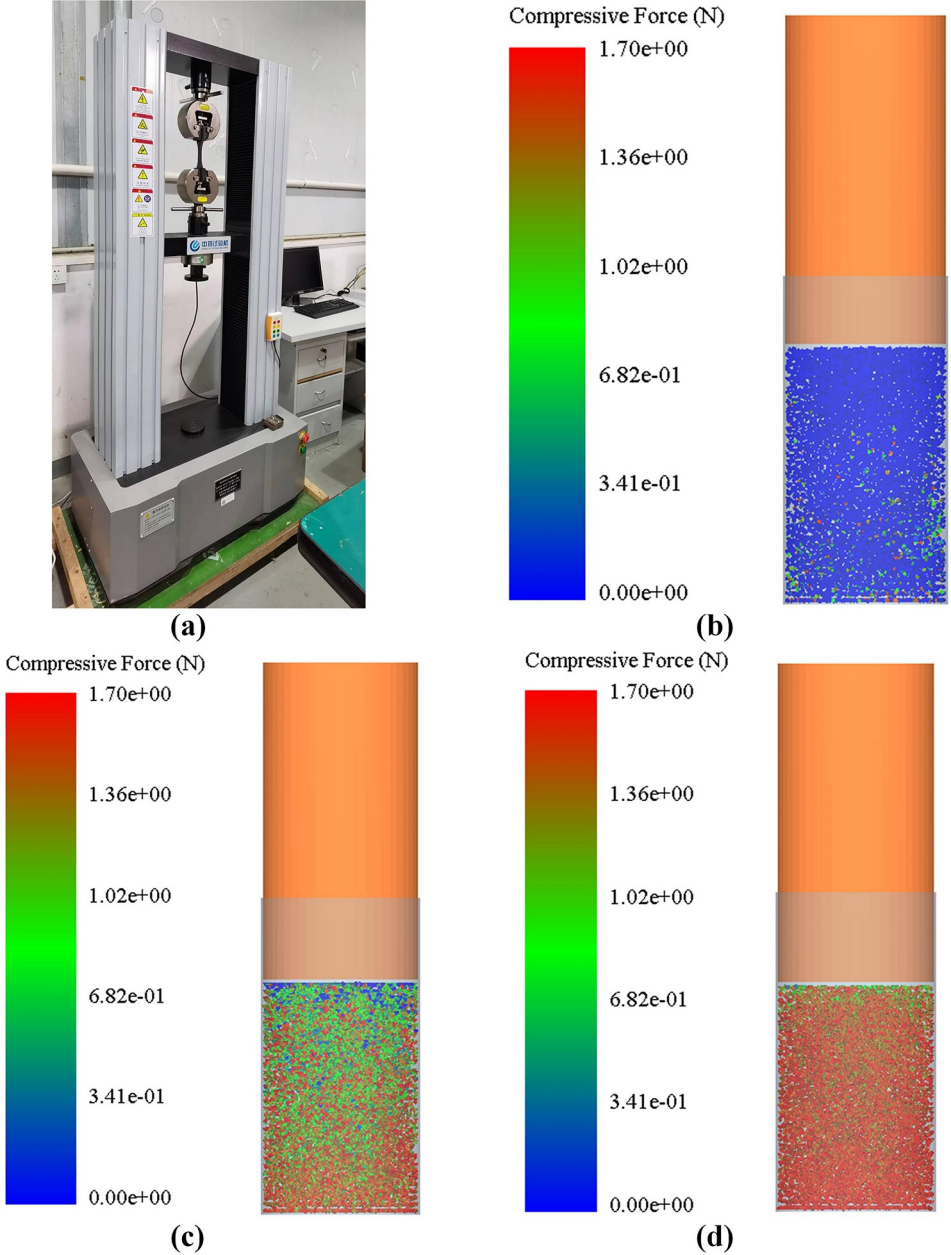
Powder particle compression experiment and EDEM simulation. (a) Compression experiment; (b) simulation initial state; (c) BF gradual compression; (d) BF compression state.

1. The simulation verification of the AR using the IM and the SCM was conducted, and the test results are shown in S1 Table. The relative error between the simulated and actual values of the different bamboo powders is less than 2.3%. Due to the limitations of computational resolution, selection of time steps, and approximation properties of simulation algorithms, DEMs may introduce additional errors when dealing with complex interparticle interactions. In addition, the nonlinear characteristics of particle dynamics, such as the instantaneous dynamic response in simulations, may have a significant impact on the predicted value of the angle of rest.
2. An experimental study on the compression of BF mold holes [12] was designed, and the results were compared with the results of compression displacement-extrusion pressure changes. A mold hole with a diameter of 20 mm and a pressure rod model were established for import into the EDEM. When the particle height is 60 mm, the pressure rod is uniformly pressed at a speed of 10 mm/min. After simulation, the pressure data during the motion process are exported through a post-processing module, as shown in Fig 10. The same size mold hole and pressure bar model were placed in the universal mechanical testing machine, BF particles of the same height were injected, and start the universal testing machine was started after adjusting the mold hole and pressure bar model axis to be in the same position, and the compression displacement-extrusion pressure data were synchronously exported.

**Fig 11:**
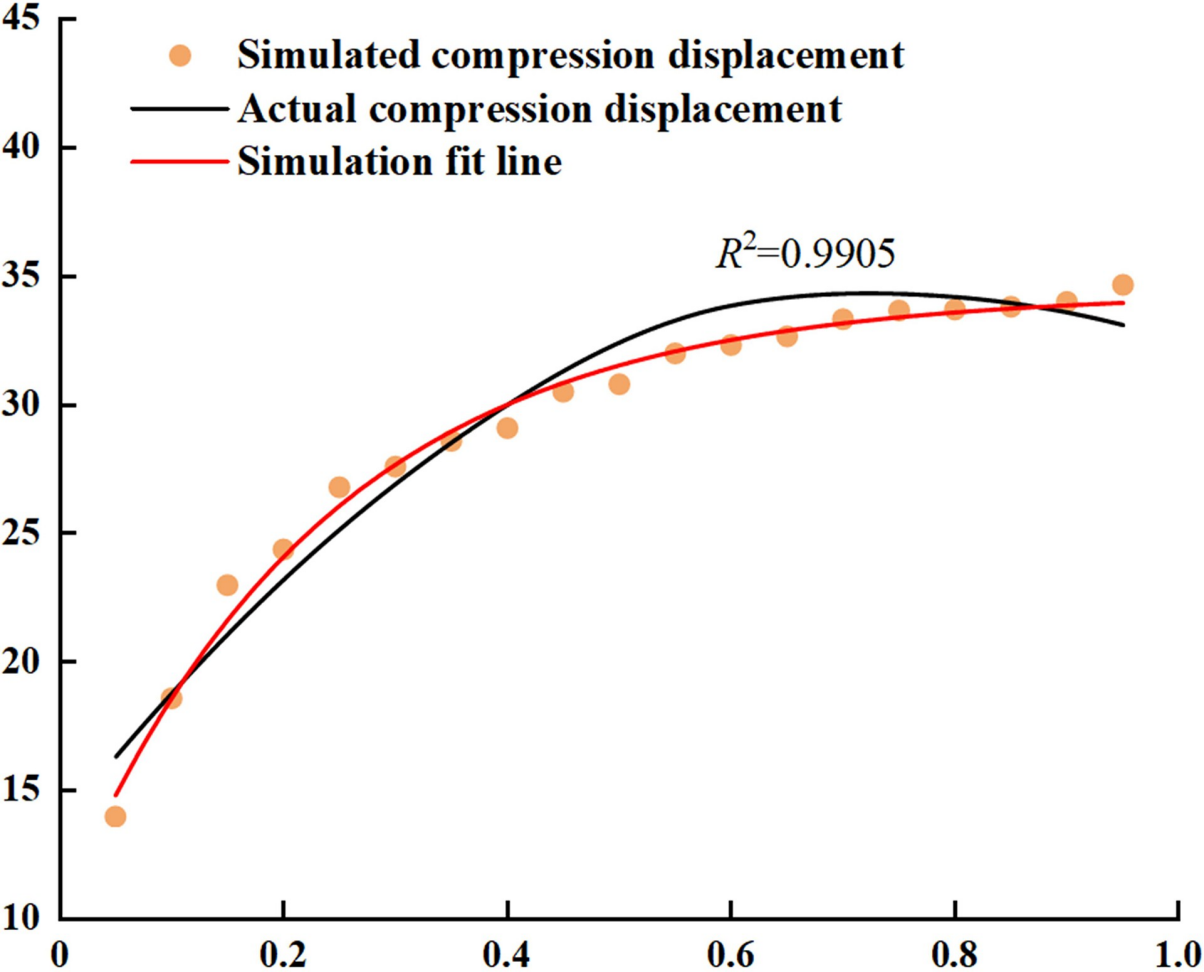
Comparative analysis of compressive displacement with BF particles.

The displacement extrusion pressure curves in the simulation and actual experimental compression tests are shown in Fig 11. In the initial stage of BF compression, the BF is in a loose state with significant displacement changes; As the powder is compressed and compacted, the displacement change gradually decreases. The maximum compression displacement and compression ratio obtained from the simulation are 34.81 mm and 0.477, respectively, which are close to the actual values of 34.77 mm and 0.461 respectively. In the simulation and actual experimental compression tests, the displacement is related to the density of the BF. When the compression force is constant, the density of the BF decreases with increasing particle size. The smaller the particle size is, the larger the surface area and contact area, and the smaller the compression displacement of the BF particles. At the same time, there are more contacts between particles, and the closer the arrangement is, the better the structural stability, and the higher the density. In addition, the magnitude of the compression force on internal particles also decreases with increasing particle size, indicating that the smaller the particle size is, the greater the contact force between particles, the smaller the compression displacement of BF particles, and the less likely they are to become loose. The analysis shows that in the simulation experiment, the diameter of the BF particles is always 1 mm, while in the actual experiment, there are differences in the size of the BF particles due to processing technology issues. Therefore, it caused errors in the simulation and practical experiments.

## 4. Conclusions

There is a lack of accurate discrete element models and parameters in the research and development of filling, drying and other processing equipment for deep processing bamboo products, such as BF. Due to the small particle size of BFs, it is difficult to simulate BF particles using traditional methods. This paper proposes a BF discrete element contact parameter calibration method based on dimensional analysis and a BP neural network. It can be concluded that:

Using particle scaling theory and dimensional analysis methods, the average particle size of the BFs was increased to 1 mm. The discrete element parameters of the 5 types of BFs to be measured were used as input layers, and the ARs obtained from the two measurement methods were used as output layers. fifty groups were randomly selected using MATLAB for EDEM simulation; The BP neural network model was used to train the simulation results, and an ideal neural network model was obtained to predict the discrete element parameters of different BFs. The predicted output of the established neural network model can reach the expected output value. AR and compression experiments were conducted under calibrated parameters, and the simulated AR was compared with the physical experimental values using the calibrated parameters. The relative error between the two was less than 2.3%. According to the BF compression test, the simulated maximum compression displacement and compression ratio were 34.81 mm and 0.477, which were close to the actual measured values of 34.77 mm and 0.461, respectively, verifying the accuracy of the neural network prediction model. This method accurately obtains the discrete element contact parameters for BF, and the results provide a reference for simulation research on BF processing and related equipment development.

## Supporting information

S1 Table Verification of discrete element contact parameters. (DOCX)

S2 Table The relevant data of simulation experiment. (DOCX)
