# Supplementary material for: Calibration of discrete meta-parameters of bamboo flour based on magnitude analysis and BP neural network
Source: PLoS One. 2024 Oct 22;19(10):e0308019. doi: 10.1371/journal.pone.0308019 (PMC11495593; doi:10.1371/journal.pone.0308019)
Supplement: S1 Table — (DOCX) [file pone.0308019.s001.docx]

**S1 Table . Verification of discrete element contact parameters.**

| **Type** | ***α*_11_** | | | ***φ*_22_** | | |
| --- | --- | --- | --- | --- | --- | --- |
|  | *x*_t_ | *x*_s_ | δ/% | *x*_t_ | *x*_s_ | δ/% |
| Z_1_ | 33.90 | 34.42 | 1.50 | 50.12 | 49.20 | 1.80 |
| Z_2_ | 39.10 | 39.99 | 2.30 | 54.91 | 56.11 | 2.20 |
| Z_3_ | 35.96 | 35.64 | 0.90 | 50.84 | 51.23 | 0.80 |
| Z_4_ | 38.10 | 37.60 | 1.30 | 56.93 | 56.26 | 1.20 |
| Z_5_ | 43.92 | 44.77 | 1.90 | 52.00 | 53.02 | 2.00 |
