# Supplementary material for: Calibration of discrete meta-parameters of bamboo flour based on magnitude analysis and BP neural network
Source: PLoS One. 2024 Oct 22;19(10):e0308019. doi: 10.1371/journal.pone.0308019 (PMC11495593; doi:10.1371/journal.pone.0308019)
Supplement: S2 Table — (DOCX) [file pone.0308019.s002.docx]

**S2 Table. The relevant data of simulation experiment.**

| **No** | **Discrete element simulation parameters** | | | | | | | | |
| --- | --- | --- | --- | --- | --- | --- | --- | --- | --- |
|  | ***x*_1_** | ***x*_2_** | ***x*_3_** | ***x*_4_** | ***x*_5_** | ***x*_6_** | ***x*_7_** | ***α*_11_** | ***φ*_22_** |
| **1** | 0.51 | 0.50 | 0.23 | 0.67 | 0.65 | 0.80 | 0.02 | 32.31 | 58.09 |
| **2** | 0.30 | 0.40 | 0.05 | 0.37 | 0.33 | 0.20 | 0.01 | 23.02 | 35.56 |
| **3** | 0.47 | 0.74 | 0.05 | 0.32 | 0.55 | 0.38 | 0.02 | 23.50 | 39.10 |
| **4** | 0.15 | 0.40 | 0.13 | 0.15 | 0.33 | 0.20 | 0.02 | 27.65 | 44.42 |
| **5** | 0.15 | 0.20 | 0.05 | 0.15 | 0.20 | 0.10 | 0.01 | 18.39 | 33.25 |
| **6** | 0.60 | 0.20 | 0.05 | 0.15 | 0.70 | 0.10 | 0.05 | 27.22 | 47.51 |
| **7** | 0.30 | 0.20 | 0.05 | 0.15 | 0.33 | 0.10 | 0.02 | 24.72 | 37.26 |
| **8** | 0.39 | 0.68 | 0.26 | 0.73 | 0.40 | 0.10 | 0.04 | 34.25 | 55.58 |
| **9** | 0.30 | 0.40 | 0.13 | 0.15 | 0.20 | 0.10 | 0.02 | 23.19 | 42.15 |
| **10** | 0.15 | 0.20 | 0.05 | 0.37 | 0.20 | 0.20 | 0.02 | 20.29 | 34.76 |
| **11** | 0.30 | 0.20 | 0.13 | 0.37 | 0.20 | 0.20 | 0.02 | 24.46 | 43.26 |
| **12** | 0.15 | 0.20 | 0.13 | 0.15 | 0.33 | 0.20 | 0.01 | 28.67 | 43.83 |
| **13** | 0.30 | 0.20 | 0.13 | 0.37 | 0.33 | 0.10 | 0.01 | 27.65 | 42.70 |
| **14** | 0.23 | 0.30 | 0.10 | 0.27 | 0.26 | 0.15 | 0.02 | 25.35 | 39.10 |
| **15** | 0.15 | 0.40 | 0.13 | 0.37 | 0.20 | 0.10 | 0.01 | 21.01 | 37.26 |
| **16** | 0.19 | 0.26 | 0.35 | 0.55 | 0.35 | 0.59 | 0.01 | 35.95 | 64.53 |
| **17** | 0.15 | 0.40 | 0.05 | 0.37 | 0.33 | 0.10 | 0.02 | 23.65 | 36.39 |
| **18** | 0.30 | 0.40 | 0.05 | 0.15 | 0.20 | 0.20 | 0.01 | 18.19 | 33.99 |
| **19** | 0.60 | 0.40 | 0.13 | 0.37 | 0.58 | 0.20 | 0.04 | 29.07 | 50.96 |
| **20** | 0.30 | 0.40 | 0.28 | 0.37 | 0.58 | 0.40 | 0.02 | 35.24 | 59.85 |
| **21** | 0.60 | 0.40 | 0.28 | 0.80 | 0.33 | 0.40 | 0.04 | 36.39 | 60.76 |
| **22** | 0.45 | 0.60 | 0.20 | 0.58 | 0.45 | 0.30 | 0.03 | 31.98 | 52.44 |
| **23** | 0.30 | 0.40 | 0.13 | 0.80 | 0.33 | 0.40 | 0.04 | 29.59 | 48.86 |
| **24** | 0.15 | 0.20 | 0.05 | 0.80 | 0.20 | 0.80 | 0.05 | 22.33 | 42.15 |
| **25** | 0.30 | 0.80 | 0.28 | 0.37 | 0.58 | 0.40 | 0.04 | 35.69 | 58.96 |
| **26** | 0.60 | 0.80 | 0.13 | 0.37 | 0.33 | 0.40 | 0.02 | 28.30 | 44.42 |
| **27** | 0.60 | 0.40 | 0.28 | 0.80 | 0.58 | 0.20 | 0.02 | 34.01 | 53.98 |
| **28** | 0.15 | 0.56 | 0.17 | 0.21 | 0.30 | 0.17 | 0.03 | 29.28 | 58.09 |
| **29** | 0.60 | 0.80 | 0.13 | 0.80 | 0.58 | 0.40 | 0.02 | 27.58 | 46.87 |
| **30** | 0.30 | 0.40 | 0.13 | 0.37 | 0.33 | 0.20 | 0.02 | 27.22 | 43.26 |
| **31** | 0.30 | 0.80 | 0.13 | 0.80 | 0.58 | 0.20 | 0.04 | 29.07 | 52.44 |
| **32** | 0.35 | 0.80 | 0.58 | 0.27 | 0.45 | 0.66 | 0.04 | 42.01 | 90.00 |
| **33** | 0.60 | 0.80 | 0.28 | 0.37 | 0.33 | 0.20 | 0.04 | 36.19 | 72.78 |
| **34** | 0.30 | 0.80 | 0.28 | 0.80 | 0.33 | 0.20 | 0.02 | 33.50 | 56.40 |
| **35** | 0.60 | 0.80 | 0.05 | 0.80 | 0.70 | 0.80 | 0.01 | 23.32 | 39.10 |
| **36** | 0.27 | 0.20 | 0.11 | 0.61 | 0.50 | 0.24 | 0.04 | 31.24 | 52.44 |
| **37** | 0.60 | 0.20 | 0.35 | 0.80 | 0.70 | 0.10 | 0.01 | 36.19 | 62.61 |
| **38** | 0.15 | 0.80 | 0.05 | 0.80 | 0.70 | 0.10 | 0.05 | 23.32 | 44.99 |
| **39** | 0.38 | 0.50 | 0.20 | 0.48 | 0.45 | 0.45 | 0.03 | 33.50 | 55.58 |
| **40** | 0.60 | 0.80 | 0.35 | 0.15 | 0.20 | 0.10 | 0.05 | 34.54 | 90.00 |
| **41** | 0.60 | 0.80 | 0.05 | 0.15 | 0.20 | 0.80 | 0.01 | 18.86 | 33.25 |
| **42** | 0.15 | 0.80 | 0.35 | 0.80 | 0.20 | 0.10 | 0.01 | 27.93 | 49.54 |
| **43** | 0.23 | 0.44 | 0.08 | 0.44 | 0.70 | 0.45 | 0.01 | 26.73 | 41.09 |
| **44** | 0.15 | 0.20 | 0.35 | 0.15 | 0.70 | 0.80 | 0.01 | 18.69 | 32.54 |
| **45** | 0.43 | 0.38 | 0.32 | 0.15 | 0.60 | 0.31 | 0.03 | 36.84 | 59.85 |
| **46** | 0.15 | 0.80 | 0.35 | 0.15 | 0.70 | 0.80 | 0.05 | 35.08 | 90.00 |
| **47** | 0.15 | 0.20 | 0.05 | 0.15 | 0.20 | 0.10 | 0.01 | 38.00 | 64.53 |
| **48** | 0.60 | 0.20 | 0.35 | 0.80 | 0.20 | 0.80 | 0.05 | 36.82 | 90.00 |
| **49** | 0.31 | 0.62 | 0.20 | 0.50 | 0.20 | 0.73 | 0.05 | 30.89 | 90.00 |
| **50** | 0.55 | 0.32 | 0.14 | 0.38 | 0.25 | 0.52 | 0.05 | 29.59 | 57.24 |
